# Comments on: Conservative Surgical Treatment of a Case of Placenta Accreta

**DOI:** 10.1055/s-0038-1675221

**Published:** 2018-10

**Authors:** Ahmed Reda

**Affiliations:** 1 Department of Obstetrics and Gynecology, Ain Shams University, Cairo, Egypt

Dear Editor,

Placenta accreta describes pathological adherence or invasion of the placenta to the myometrium. It may be a consequence of any procedure affecting the integrity of the uterine lining.^1^ The incidence is rising due to increase in the rate of cesarean delivery, which is the major risk factor. Published guidelines^2^ ^3^ ^4^ recommend delivery with planned cesarean hysterectomy and placenta left in situ, while application of conservative management must be individualized according to the patient's desire for future fertility.

In certain cases, the implementation of alternatives to standard or agreed interventions is necessary to preserve the potential for future fertility, but this may carry risk of morbidity and adverse events either from the procedure itself or due to deviation from the agreed management published in the guidelines. Such procedures should be individualized to each case according to history, clinical judgment and the patient's desire for future fertility.

Biyik et al^5^ reported a case of placenta accreta managed conservatively with segmental uterine resection, with the aim of fertility preservation. From the scenario of the presented case, it is obvious that the patient had completed her family; at the time of the surgery, the patient will be para 4, 39 years old and she requested tubal ligation, which suggests that she is not interested in future fertility. Although the authors stated that blood loss was not measured, significant hemorrhage could be detected from the change in the hemoglobin level from 10.3 g/dL preoperative to 8.5 g/dL postoperative after transfusion of 1 blood unit.

In my opinion, subjecting the patient to hemorrhagic morbidity, with added risks of blood transfusion, to pursue future fertility in a patient requesting permanent contraception—which is already performed during the same operation—is not justified.

## Notes

Authors' Reply There are limited studies on conservative treatment in cases of placenta accreta spectrum (PAS) disorder.^6^ ^7^ ^8^ ^9^ ^10^ ^11^ Unfortunately, there is no randomized controlled study that compares hysterectomy and conservative methods according to maternal morbidity, bleeding and complications in cases of PAS disorder. It is not yet possible to say that the conservative method would increase the risk of bleeding and the morbidity of the patient. It has been reported that in a limited number of studies, local/segmental resection can be tried in PAS disorder cases. Kilicci et al^8^ applied segmental resection of the anterior uterine wall to 11 cases with placenta percreta, and they reported that the mean preoperative hemoglobin value was 11.6 g/dL, and postoperatively, it was 8.5 g/dl.^8^ In our case, the placenta was on the anterior uterine wall. The hemoglobin levels were 10.3 g/dL in the preoperative period, and 8.5 g/dL in the postoperative period. Our hemogram values are similar to those of Kilicci et al.^8^ In addition, other studies have reported that transfusion was applied to the patients who had conservative treatment.^6^ ^7^ ^9^ Therefore, the fact that our transfusion application increases the morbidity in our patient is not correct, in this case. In addition, there is no evidence in the literature that patients who underwent hysterectomy present a lower transfusion rate than those who underwent conservative treatment. The patient was 39 years old and requested permanent sterilization. However, the patient's lack of fertility request does not mean that she wants hysterectomy. We think that the decision to apply the conservative method would be more appropriate according to the patient's wish, clinical situation and physician's experience. The experience of the physician in making the decision about the most appropriate surgical technique is emphasized in various studies.^12^
As a result, that the practice of conservative treatment in women who do not expect fertility increases the morbidity of the patient reflects the personal opinion of the critic. There is not enough evidence in the literature to support this idea.
